# Amelioration of autoimmune arthritis by adoptive transfer of Foxp3-expressing regulatory B cells is associated with the Treg/Th17 cell balance

**DOI:** 10.1186/s12967-016-0940-7

**Published:** 2016-06-28

**Authors:** Mi Kyung Park, Young Ok Jung, Seon-Yeong Lee, Seung Hoon Lee, Yu Jung Heo, Eun Kyung Kim, Hye Jwa Oh, Young Mee Moon, Hye-Jin Son, Min Jung Park, Sung Hwan Park, Ho Youn Kim, Mi La Cho, Jun Ki Min

**Affiliations:** The Rheumatism Research Center, Catholic Research Institute of Medical Science, The Catholic University of Korea, 505 Banpo-dong, Seocho-gu, Seoul, 137-040 South Korea; Division of Rheumatology, Department of Internal Medicine, Hallym University Kang-Nam Sacred Heart Hospital, Seoul, South Korea; Division of Rheumatology, Department of Internal Medicine, College of Medicine, The Catholic University of Korea, Seoul, South Korea; Bucheon St. Mary’s Hospital, Division of Rheumatology, Department of Internal Medicine, College of Medicine, The Catholic University of Korea, 327 Sosa-ro, Wonmi-gu, Bucheon, Gyeonggi-do 420-717 South Korea; Division of Rheumatology, Department of Internal Medicine, College of Medicine, Holy Family Hospital, Rheumatism Research Center (RhRC), Catholic Research Institute of Medical Science, The Catholic University of Korea, Seoul, South Korea

**Keywords:** Foxp3, Regulatory B cell, Th17, Arthritis

## Abstract

**Background:**

Foxp3 is a key regulator of the development and function of regulatory T cells (Tregs), and its expression is thought to be T cell-restricted. We found that B cells in mice can express Foxp3 and B cells expressing Foxp3 may play a role in preventing the development of collagen-induced arthritis (CIA) in DBA/1J mice.

**Methods:**

Foxp3 expression was modulated in CD19^+^ B cells by transfection with shRNA or using an over-expression construct. In addition, Foxp3-transfected B cells were adoptively transferred to CIA mice. We found that LPS or anti-IgM stimulation induced Foxp3 expression in B cells. Foxp3-expressing B cells were found in the spleens of mice.

**Results:**

Over-expression of Foxp3 conferred a contact-dependent suppressive ability on proliferation of responder T cells. Down-regulation of Foxp3 by shRNA caused a profound induction in proliferation of responder T cells. Adoptive transfer of Foxp3^+^CD19^+^ B cells attenuated the clinical symptoms of CIA significantly with concomitant suppression of IL-17 production and enhancement of Foxp3 expression in CD4^+^ T cells from splenocytes.

**Conclusion:**

Our data indicate that Foxp3 expression is not restricted to T cells. The expression of Foxp3 in B cells is critical for the immunoregulation of T cells and limits autoimmunity in a mouse model.

## Background

B cells exert a variety of immune functions, including the production of immunoglobulins (Igs) and cytokines, the presentation of antigens, and the regulation of dendritic cells [1–4]. B cells are generally considered to positively regulate immune responses by producing antigen(Ag)-specific antibodies (Abs) and inducing CD4^+^ T cell activation [5]. B cells are involved in the development of several autoimmune disorders through the production of pathogenic Igs [6, 7]. Especially, immune-regulatory roles of B cells in autoimmune diseases have been reported that specific B cell subsets regulate immune responses and participate in the induction of immune tolerance [8, 9].

The existence of B cells with regulatory properties has been widely reported [10–14]. Several studies have shown that absence of B cells exacerbated pathologic inflammatory responses in autoimmune diseases [12, 14]. B cell-deficient (μMT) mice lacked the capacity to resolve inflammation in Experimental Autoimmune Encephalomyelitis [1]. Mizoguchi and colleagues introduced the term ‘regulatory B cells (Bregs)’ to designate B cells with negative regulatory properties [15]. Experimental studies have demonstrated that the absence or loss of Bregs exacerbates symptoms in several experimental autoimmune disease model including collagen-induced arthritis (CIA) [15–21]. Additionally, Bregs showed therapeutic properties in autoimmune arthritis mice models [18, 22].

Rheumatoid arthritis (RA) is a debilitating autoimmune disease characterized by chronic inflammation and destruction of the joints has been considered to be a Th1 and/or Th17-mediated disease. However, B cells also play important roles in the pathogenesis of RA. B cells present within the synovial membrane of affected joints are involved directly in sustained inflammation in the rheumatoid synovium [3], and play a critical role in the synthesis of rheumatoid factor (RF) [23]. The therapeutic success of B cell depletion using a mAb against the B-cell surface molecule CD20 (Rituximab; RTX) has brought in a renewed focus on the role of B cells in the pathogenesis and control of RA and other autoimmune diseases [24, 25]. Interestingly, regulatory B cells have also been proposed to play a role in the K/BxN arthritis mouse model, a model in which Igs are required for disease development. Furthermore, the number of regulatory B cells was negatively correlated with disease activity in new onset RA patients [26]. Several different Breg subsets have now been identified and characterized phenotypically as CD5^+^ B-1a, CD1d^+^ marginal zone B cells, transitional-2-marginal zone precursor B cells, and CD1d^hi^CD5^+^CD19^hi^ in mouse models.

The transcription factor Foxp3 is a master regulator of Tregs, controlling their development and function. A role for Foxp3 in maintaining self tolerance has been shown in scurfy mice, and in patients with immunodysregulation, polyendocrinopathy, enteropathy, and X-linked (IPEX) syndrome as the causative genetic anomaly that results in severe autoimmune diseases [27–29]. The expression of Foxp3 in conventional T cells confers suppressive activity and induces the expression of associated molecules such as CD25, cytotoxic T lymphocyte antigen 4 (CTLA4), and glucocorticoid-induced TNF receptor-related protein (GITR) [30–32]. These findings suggest that B cells with suppressive activity may also express Foxp3. Foxp3 expressing CD19(+)CD5(+) B cell population was identified in human peripheral blood mononuclear cells and regulatory properties of this cell type was proposed [33]. The Foxp3 expressing regulatory B cells were identified as strong suppressors in milk allergy in human.

In the present study, we investigated the existence of Foxp3-expressing B cells, and their regulatory roles in mice arthritis model, by testing whether they could regulate the proliferation of effector T cells in vitro through a cell-to-cell contact-dependent mechanism. Furthermore, we found a therapeutic effect of Foxp3^+^ B cells in autoimmune arthritis by performing cell transfer studies in CIA mice, an animal model of RA. We observed that the Foxp3^+^ B cells showed a strong suppressive effect against arthritis in CIA mice.

## Methods

### Mice

Male DBA/1J mice aged 6–8 weeks were purchased from the Charles River Laboratory (Yokohama, Japan). Mice were housed in groups of 10 and maintained at a mean temperature of 21 °C (±2 °C) on a 12-h light/12-h dark cycle, with food and water available ad libitum. All experimental procedures were examined and approved by the Animal Research Ethics Committee of the Catholic University of Korea (permit number: CUMC-2009-0044-01), which conforms to all National Institutes of Health of the USA guidelines. All surgeries were performed under isoflurane anesthesia, and all efforts were made to minimize suffering.

### Cell preparation and culture

The A20 cell line (mouse B cell lymphoma) was purchased from the American Type Culture Collection (ATCC; Rockville, MD, USA). Spleens were collected for cell preparations from DBA/1J or arthritis mice. To purify CD19^+^ B cells or CD4^+^ T cells, splenocytes were incubated with CD19 or CD4 microbeads (Miltenyi Biotec, Auburn, CA, USA) and isolated on MACS separation columns. B cells or T cells determined by staining with FITC-labeled anti-CD19 mAb or PE-labeled anti-CD4 mAb, respectively (BD Biosciences Pharmingen, San Diego, CA, USA). These cells were >98 % purity. CD19^+^ B cells or A20 cells were cultured with various stimuli, such as 10 µg/ml lipopolysaccharide (LPS; Sigma-Aldrich, St. Louis, MO, USA) or 10 µg/ml anti-IgM (Jackson ImmunoResearch, West Grove, PA, USA) for 72 h.

### Flow cytometric analysis

CD19^+^ B cells were resuspended in 4 % BSA flow buffer and blocked with CD16/CD32 Fc antibody (BD Pharmingen). Cells were incubated on ice for 30 min with FITC-labeled anti-CD19 mAb or PerCP cy5.5-labeled anti-CD4 mAb (all from eBioscience, San Diego, CA, USA). For intracellular staining of Foxp3 and CTLA4, cells were fixed, permeabilized, and stained with FITC- or PE-labeled anti-Foxp3 mAb and/or PE-labeled anti-CTLA4 mAb (all from eBioscience). Finally, cells were analyzed using a FACSCalibur (Becton–Dickinson, San Jose, CA, USA). Cells that stained positively for CD4, CD19, CD25, IL-17 and Foxp3 were counted visually at higher magnification by four individuals, and the mean values were presented in the form of a graph.

### Transfection of CD19^+^ B cells

The Foxp3 open reading frame (ORF) was codon optimized for mammalian codon usage and was synthesized by Genscript Corp. (Piscataway, NJ, USA). The Foxp3 ORF was subcloned into the pcDNA3.1 (+) mammalian expression vector (Invitrogen, Carlsbad, CA, USA), digested with Hindlll and Xho l sites. The construct was verified by DNA sequencing (Macrogen, Seoul, Korea). Foxp3-specific targeting short hairpin RNA (shRNA) was purchased from Santa Cruz Biotechnology (Santa Cruz, CA, USA). For transfection, splenic CD19^+^ B cells were seeded in 24-well plates. Cells were transfected with 1.5 µg of DNA using poly-MAG and Magneto FACTOR plates (Chemicell GmbH, Berlin, Germany), according to the manufacturer’s instructions. Cell viability was assessed using the Cell Counting kit-8 (CCK-8, Dojindo Laboratories, Kumamoto, Japan).

### RT-PCR analysis of mRNA expression

Total RNA was extracted using TRIZOL® Reagent (Invitrogen) and cDNA synthesis was performed using oligo-dT primers and AMV reverse transcriptase (Promega, Mannheim, Germany), according to the manufacturer’s instructions. PCR amplification of cDNA aliquots was performed by addition of 2.5 mM dNTPs, 2.5 U *Taq* DNA polymerase (Takara, Shiga, Japan), and 0.25 µM sense and antisense primers. The following sense and antisense primers were used: mice Foxp3, 5′- CCC AGG AAA GAC AGC AAC CTT-3′ (sense) and 5′- TTC TCA CAA CCA GGC CAC TTG-3′ (antisense), and mice β-actin, 5′-GAA ATC GTG CGT GAC ATC AAA G-3′ (sense) and 5′-TGT AGT TTC ATG GAT GCC ACA G-3′ (antisense). Reactions occurred in a DNA thermal cycler (PerkinElmer, Norwalk, CT) and comprised 30–35 cycles of 94 °C for 30 s, 60 °C for 30 s, and 72 °C for 30 s. PCR products were run on a 2.5 % agarose gel and gels and visualized under ultraviolet light using a Gel-doc 1000 (Bio-Rad, Hercules, CA, USA).

### Western blot analysis

CD19^+^ B cell lysates were denatured in SDS, resolved by 10 % SDS-PAGE, and transferred to polyvinylidene fluoride membranes (Amersham Pharmacia, NJ, USA). Membranes were pre-incubated with 5 % skimmed milk in TBS for 2 h at room temperature. Primary Abs directed against Foxp3 (Santa Cruz Biotechnology), diluted 1/200 in blocking buffer (5 % skimmed milk in TBS), were then added and the samples incubated overnight at 4 °C. After the samples were washed for four times in TBST, HRP-conjugated secondary Abs were added and incubated for 1 h at room temperature. Finally, membranes were washed in TBST and the hybridized bands were detected with an ECL detection kit (Pierce, Rockford, IL, USA).

### Confocal immunofluorescence assay

For confocal staining, 7-µm sections of spleen tissue was fixed in acetone and blocked with 20 % FCS/PBS. After washing, slides were stained using PE or FITC-labeled anti-Foxp3, PE-labeled anti-IL-17, biotinylated anti-CD19, APC-labeled anti-CD25 and FITC, or PerCP cy5.5-labeled anti-CD4, followed by streptavidin-FITC. After being washed, slides were mounted and visualized using a Zeiss microscope (LSM 510 Meta; Carl Zeiss, Oberkochen, Germany). Results were mean value of 4 sections in spleens from 3 animals. We presented representative figure.

### Suppression assay

CD4^+^CD25^−^ T cells were isolated from spleens of arthritic mice by magnetic bead cell sorting using an untouched CD4^+^ T Cell Isolation Kit II and CD25 Microbeads (all from Miltenyi Biotec, Bergisch Gladbach, Germany), according to the manufacturer’s instructions. To assess the suppressive activities of Foxp3-transfected CD19^+^ B cells, CD4^+^CD25^−^responder T cells (5 × 10^4^/well) were cultured with a 1:1 ratio of shRNA or Foxp3-transfected CD19^+^ B cells (which were stimulated with LPS or anti-IgM) in the presence or absence of bovine type II collagen (CII) (Chondrex Inc., Redmond, WA, USA), in a 100 ng/ml anti-CD3-coated 96-well plate. In some cases, Foxp3-transfected CD19^+^ B cells were placed in the inner transwell chamber. During the last 16 h of culture, cells were pulsed with ^3^H-thymidine (1 μCi/well; MP Biomedicals, Seven Hills, Australia). Cells were assessed for thymidine incorporation in a Microbeta counter (Wallac Oy 1450 MicroBeta; Wallac, Melbourne, Australia).

### Induction and clinical assessment of arthritis

CII was dissolved in 0.1 M acetic acid solution (2 mg/ml) by gentle rotation at 4 °C overnight. Mice were injected intradermally at the base of the tail with 100 μg CII emulsified with an equal volume of CFA containing 2 mg/ml *Mycobacterium tuberculosis* (Chondrex Inc). On day 14, a second injection of CII in IFA was administered. Arthritic indices were evaluated three times weekly by three or more independent investigators until 9 weeks after the first immunization. The scale of the arthritis index ranged from 0 to 4. Scores were defined as follows: 0, no evidence of erythema or swelling; 1, erythema and mild swelling confined to the mid-foot (tarsal part) or ankle joint; 2, erythema and moderate swelling extending from the ankle to the mid-foot; 3, erythema and moderate swelling extending from the ankle to the metatarsal joints; 4, erythema and severe swelling encompassing the ankle, foot, and digits [34].

### Histological assessment of arthritis

At sacrifice, knee joints (mid-tibia to mid-femur) were harvested, and the joints were fixed overnight in 4 % paraformaldehyde Decalcified limbs were embedded in paraffin and sectioned to a 7-µm thickness. Tissues were stained with hematoxylin-eosin (H&E), Toluidine blue, and Safranin O. For histological evaluation of arthritis, sections were evaluated in a blind manner. The scores were evaluated as described previously [35].

### Adoptive transfer

Splenic CD19^+^ B cells of naïve mice were purified with magnetic beads (Miltenyi Biotec). Purified spleen B cells were transfected with mock or Foxp3 over-expression construct and cultured with LPS for 72 h. Purified 10 × 10^6^ Foxp3-transfected CD19^+^ B cells were suspended in a total of 200 μl saline and transferred intravenously into mice on days 7 and 28 after CII immunization.

### Statistical analysis

Experimental values presented are the means ± SD. Student’s *t* tests were performed to calculate the statistical differences between means of different variables, and *P* values less than 0.05 (two-tailed) were considered significant.

## Results

### LPS and IgM stimulation induced Foxp3 expression in mouse B cells

**Fig. 1:**
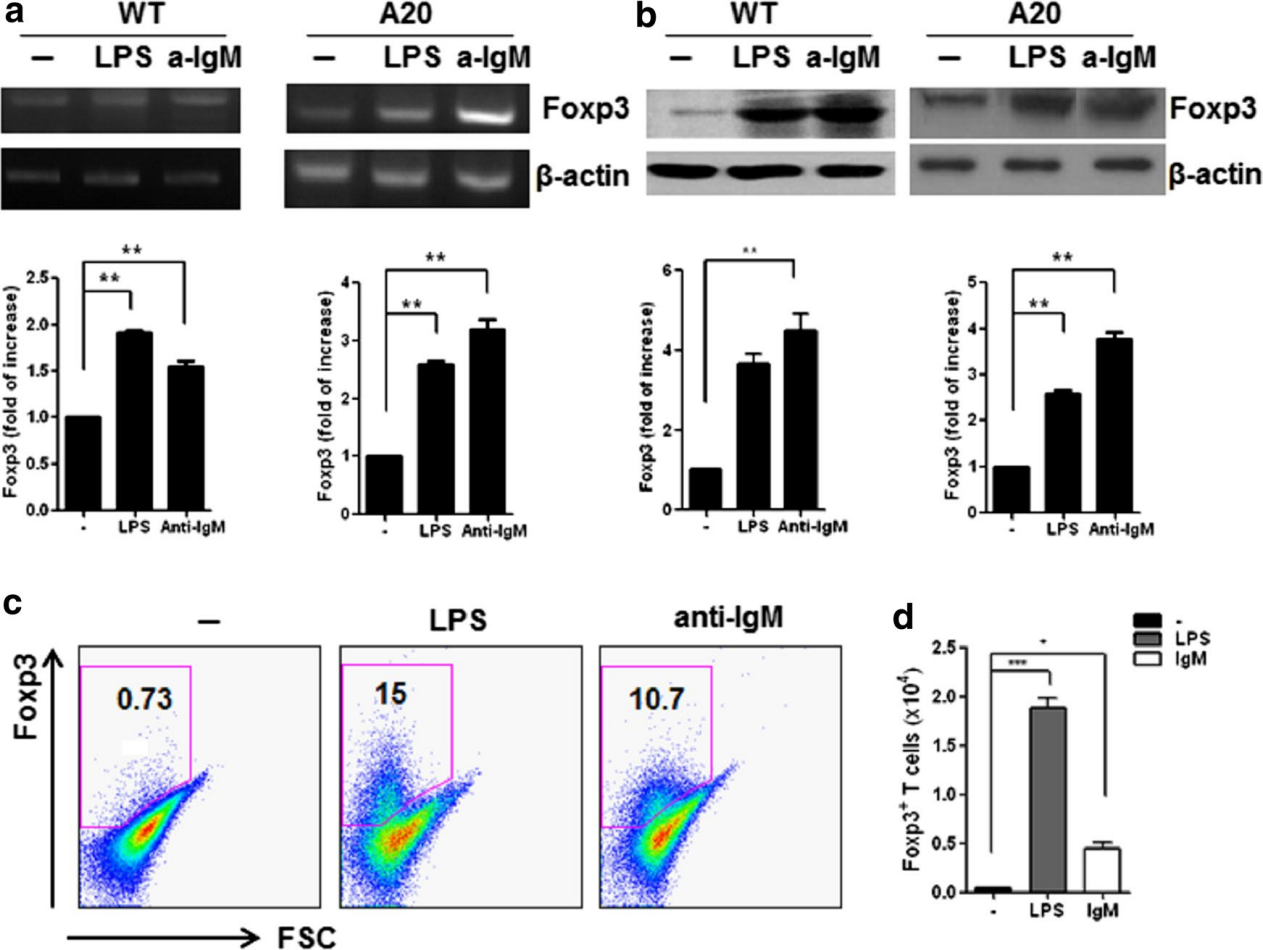
Foxp3 is expressed by LPS and anti-IgM in mouse B cells. Purified CD19^+^ B cells were isolated from spleens of 6- to 8-week-old DBA/1J mice. CD19^+^ primary B cells isolated from splenocytes of wild type mice or A20 (mouse B cell line) were stimulated with 10 µg/ml LPS, or 10 µg/ml anti-IgM for 72 h. Foxp3 **a** mRNA and **b** protein levels were determined by RT-PCR and western blot, respectively. The optical densities of Foxp3 bands were normalized to those of β-actin. The data represent one of three similar experiments. *Bar graphs* show mean ± SD. Data are representative of three independent experiments. Significant differences between means of cells cultured in media alone and stimulated cultures are indicated: *p < 0.05, **p < 0.01 compared with untreated cells. **c** Whole splenocytes were stained with CD19-FITC mAb before permeabilization and stained using a Foxp3-PE mAb. *Numbers* indicate Foxp3^+^ cell frequencies within CD19^+^ B cell subsets. The graph on the right represents the absolute cell numbers of Foxp3^+^ Breg cells. Data are representative of three independent experiments. **d** Confocal microscopic analysis of cultured CD4-deplected splenocytes for Foxp3 (*red*, PE), and CD19 (*green*, FITC). Representative images after LPS treatment are shown. Original magnification, ×400

To evaluate the effect of LPS and IgM on Foxp3 expression of B cells, we cultured splenic CD19^+^ B cells in the presence of LPS or IgM for 72 h. Foxp3 expression was ascertained by PCR, immunoblotting, and flow cytometry. Increased in Foxp3 mRNA and protein expression were observed in both cultured CD19^+^ primary B cells isolated from splenocytes of wild type mice (WT), and in a mouse B cell lymphoma line (A20), after LPS or IgM mAb stimulation (Fig. 1a, b). Flow cytometric analysis of cultured B cells in media alone showed that 0.73 % of B cells expressed Foxp3. After stimulation with LPS or IgM mAb, more than 10 % of the CD19^+^ cells expressed Foxp3 (Fig. 1c). Additionally, LPS and IgM mAb induced an increased level of Foxp3 expression in CD19^+^ B cells, as determined by confocal microscopy (Fig. 1d).

### CD19^+^ Foxp3^+^ B cells exist in spleens of CIA mice

**Fig. 2:**
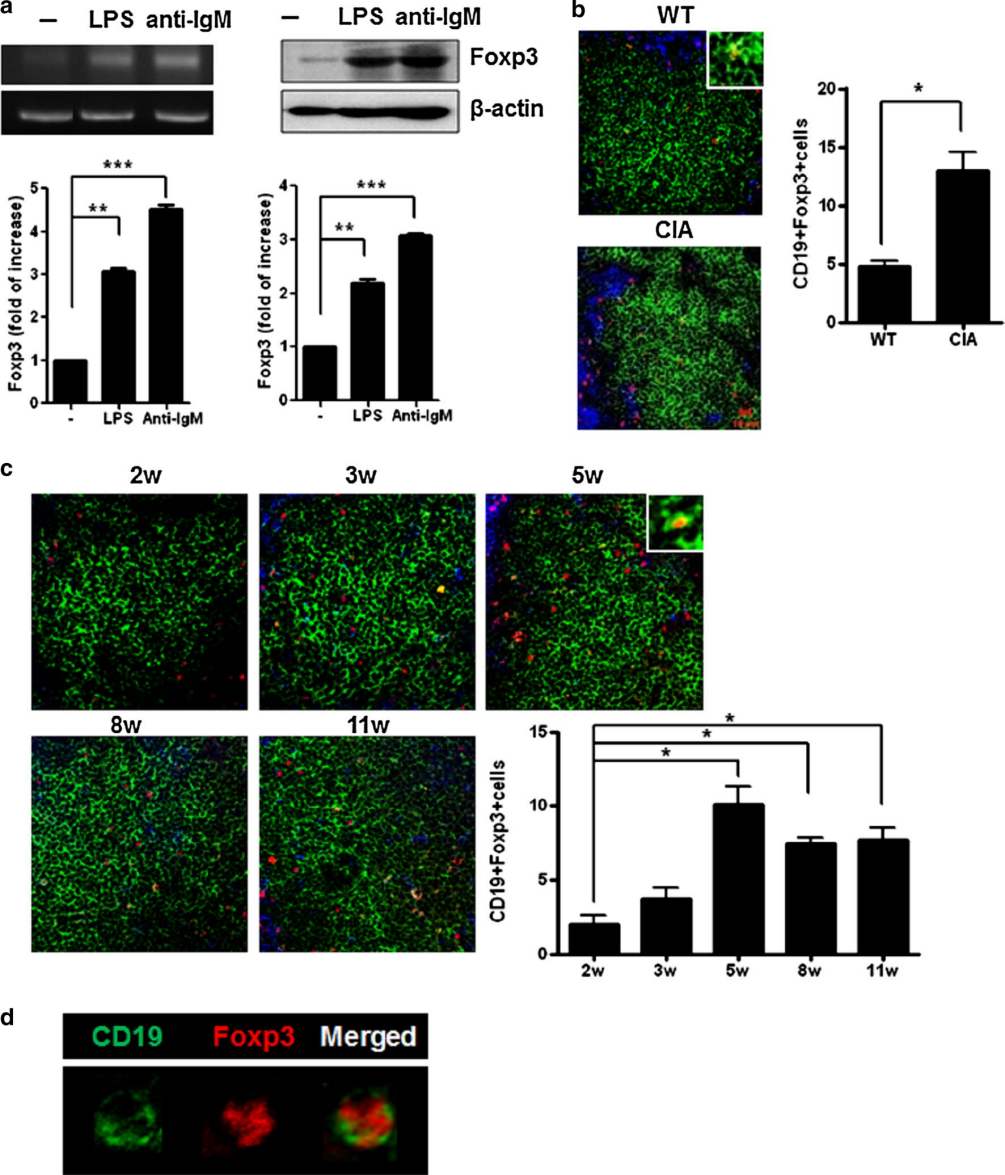
CD19^+^ Foxp3^+^ B cells exist in spleens of collagen-induced arthritis (CIA) mice. CIA was induced in 6- to 8-week-old male DBA/1J mice by intradermal immunization in the base of the tail with Cll/CFA emulsion on day 0, followed by a booster injection on day 21, as described in the “Methods” section. **a** Purified B cells isolated from the spleen of CIA mice were cultured with 10 µg/ml LPS or 10 µg/ml anti-IgM. Following 72 h of activation, Foxp3 mRNA (*left*) and protein (*right*) levels were determined by RT-PCR and Western blot, respectively. The optical densities of Foxp3 bands were normalized to those of β-actin. **b** Spleens of DBA/1J or CIA mice (5 weeks after primary CII immunization) were stained with CD19-FITC, CD4-PerCP cy5.5, and Foxp3-PE mAbs and analyzed by confocal laser microscopy. The numbers in the graphs indicate the cell count of positive cells counted microscopically. **c** Kinetic analysis of Foxp3-expressing B cells in the spleens of CIA mice. Original magnification, ×400. **d** The expression of CD19 and Foxp3 in CIA mice splenocytes. The data represent one of three similar experiments. *Bar graphs* show mean ± SD. Statistical significance was defined at *p < 0.05 compared with 3 weeks after immunization, **p < 0.01, ***p < 0.001 compared with untreated cells

Our in vitro studies suggested that stimulation with BCR or BCR-independent activators induced Foxp3^+^ in B cells. Therefore, these cells may be related to an inflammatory environment such as RA. Next, we investigated whether B cells that expressed Foxp3 were present in the spleens of CIA mice. Foxp3 mRNA and protein expression was detected in splenic B cells under basal conditions and was further up-regulated by LPS or anti-IgM stimulation (Fig. 2a). By confocal microscopy, we identified Foxp3 expression in splenic CD19^+^ B cells, although the vast majority of Foxp3^+^ cells were CD4^+^ T cells (Fig. 2b). We observed more CD19^+^Foxp3^+^ B cells in CIA than in WT DBA/1 mice, the numbers of which peaked at 5 weeks post-immunization (Fig. 2c). Foxp3^+^ B cells peaked at 5 weeks and resided in the B cell zone up to 11 weeks, although the frequency of Foxp3^+^ B cells at 8 weeks was less than at 5 weeks of immunization. Since T cell mediated immune response was occurred before B cell mediated immune response, the number of CD4^+^Foxp3^+^ cells were more than that of CD19^+^Foxp3^+^ cells in 5 weeks of immunization. We also detected CD19^+^Foxp3^+^ B cells in CIA mice (Fig. 2d). These data indicated that Foxp3-expressing CD19^+^ B cells exist naturally in the spleens of mice under inflammatory conditions such as CIA.

### Foxp3-transfected B cells have suppressor activity in vitro

**Fig. 3:**
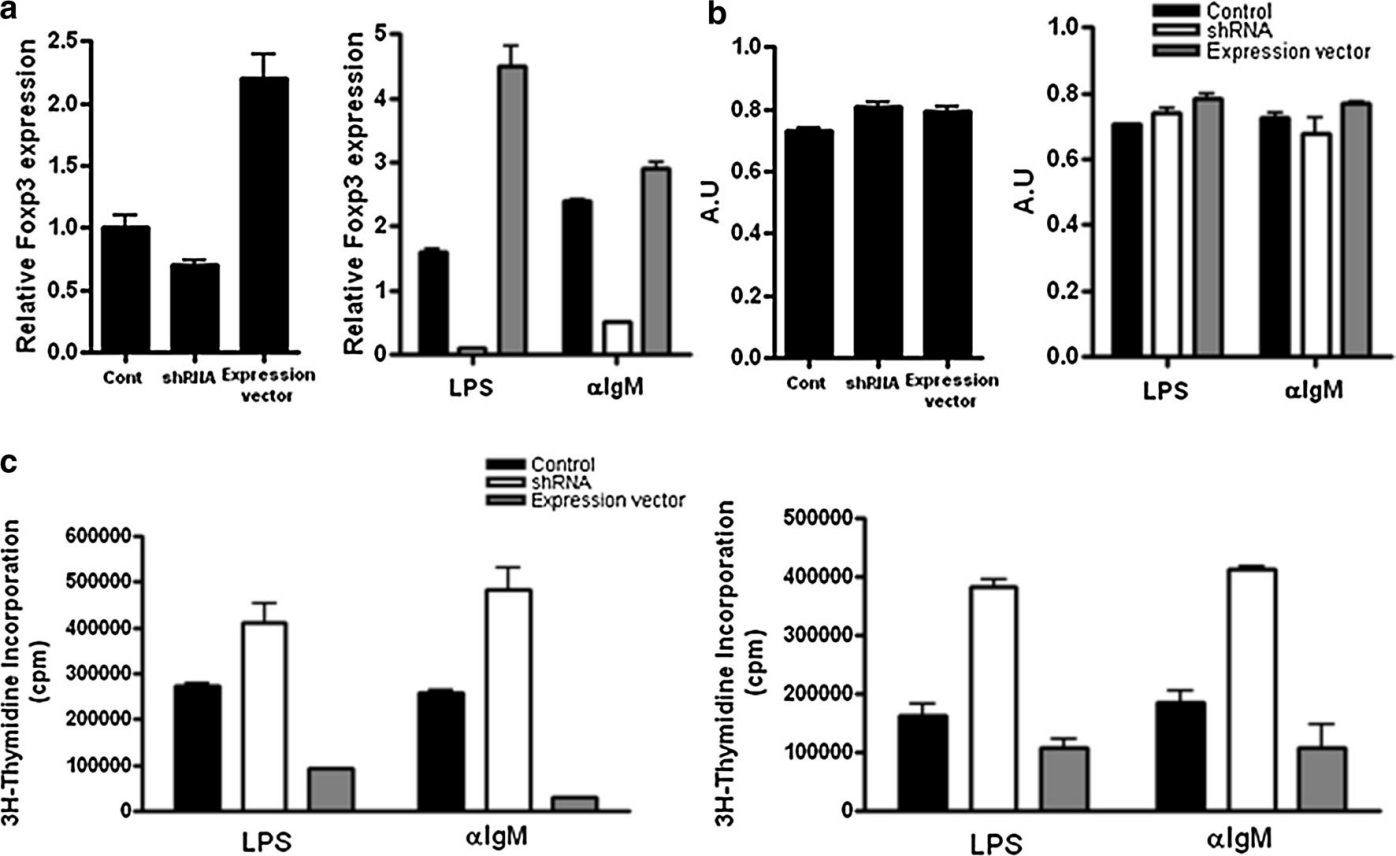
In vitro generation of regulatory B cells with suppressor activity by transfection of Foxp3. Splenic CD19^+^ B cells from DBA/1J mice were transfected with Foxp3 shRNA, a Foxp3 over-expression construct, or control plasmid DNA, and then stimulated with 10 µg/ml LPS, or 10 µg/ml anti-IgM for 72 h. **a** Relative Foxp3 mRNA levels in Foxp3 shRNA or Foxp3 over-expressing cells were determined by RT-PCR using primers specific for Foxp3 or β-actin. The relative quantity of Foxp3 was normalized to that of β-actin in each sample. **b** Cell viability of CD19^+^ B cells by MTT assay 24 h after plasmid transfection. **c** Suppression of the proliferation of responder CD4^+^CD25^−^ T cell stimulation with anti-CD3 by B cells. CD4^+^ CD25^−^ T cells isolated from the spleens of CIA mice were cultured with Foxp3 shRNA or Foxp3-transfected CD19^+^ B cells in the absence (*left panel*) or presence (*right panel*) of Cll. Proliferation was determined after 3 days of culture by ^3^H-thymidine incorporation. Cultures were harvested and cpm were determined. Values are the mean ± SD from triplicate cultures. Data represent one of the two independent mice

Foxp3 plays an essential role in the suppressive function of Treg cells. Foxp3^+^ Treg cells inhibit the activation of autoreactive T cells in an antigen-specific manner [36]. Therefore, we determined whether B cell suppressor activity depends on Foxp3 expression. CD19^+^ B cells were transfected with either Foxp3-specific shRNA, or an over-expression construct. Foxp3 over-expression led to a two-fold up-regulation compared to control upon non-stimulation and stimulation with LPS. Cells transfected with shRNA showed low levels of Foxp3 expression (Fig. 3a). Transfected cells did not undergo apoptosis (Fig. 3b). Since CIA is induced by type II collagen, we studied that B cells expressing foxp3 can regulate the proliferation of type II collagen specific T cells. CD19^+^ B cells transfected with Foxp3 efficiently suppressed the proliferation of CD4^+^ T cells stimulated with CD3 mAb in the absence or presence of CII. In contrast, Foxp3-specific shRNA-transfected cells showed no inhibitory effect on T cell proliferation (Fig. 3c). These data showed that expression of Foxp3 is induced after BCR stimulation by LPS or anti-IgM, and that the suppressive activity of CD19^+^ B cells is due to the expression of Foxp3.

### Foxp3^+^ expressing B cells-mediated cell contact-dependent suppression of T cell proliferation

**Fig. 4:**
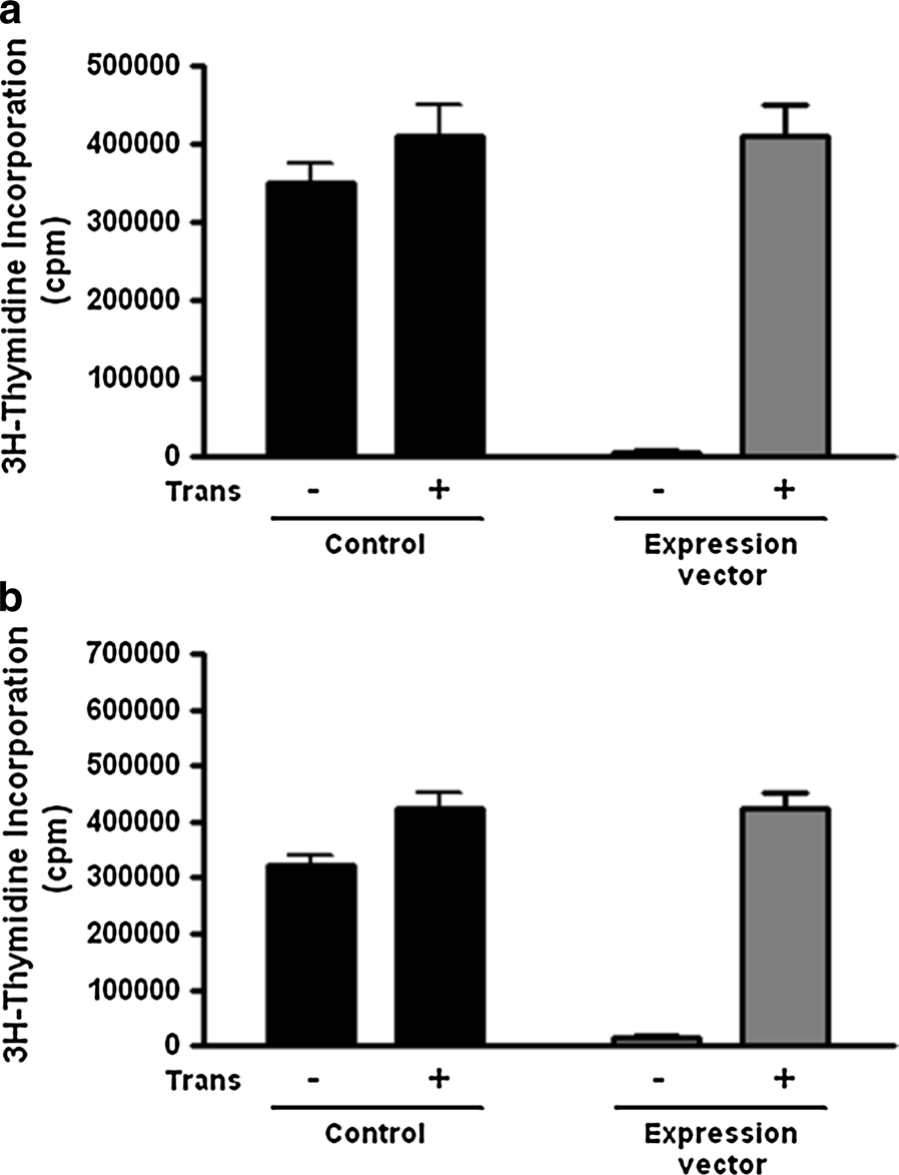
Immunosuppressive action of Foxp3-trasnfected CD19^+^ B cells was dependent on cell contact. **a**, **b** Splenic CD19^+^ B cells from DBA/1J mice were transfected with Foxp3 or control plasmid DNA and then stimulated with 10 µg/ml LPS (**a**) or 10 µg/ml anti-IgM (**b**) for 72 h. CD4^+^CD25^−^ T cells were stimulated with anti-CD3 and co-cultured with transfected B cells either directly (no chamber) or in the upper chamber of a transwell plate. Proliferation was determined after 3 days of culture by ^3^H-thymidine incorporation. Cultures were harvested and cpm were determined. Values are the mean ± SD from triplicate cultures. Data represent one of the two independent mice

To identify whether Foxp3 can reduce T cell proliferation in the cell to cell contact, we measured T cell proliferation using Foxp3 over-expression vector transfected cells. The use of an in vitro transwell assay allows confirmation of whether direct contact with Foxp3^+^CD19^+^ B cells is required for proliferation of CD4^+^CD25^−^ T cells. Responder T cells were cultured with transfected B cells either in direct contact or separated by a transwell. The suppressor activity of CD19^+^ B cells transfected with Foxp3 was lost in the absence of cell–cell contact (Fig. 4a, b). These findings suggest that the suppressor activity of Foxp3-expressing B cells occurred via cell–cell contact.

### Foxp3-transfected B cells inhibit autoimmune arthritis in mice

**Fig. 5:**
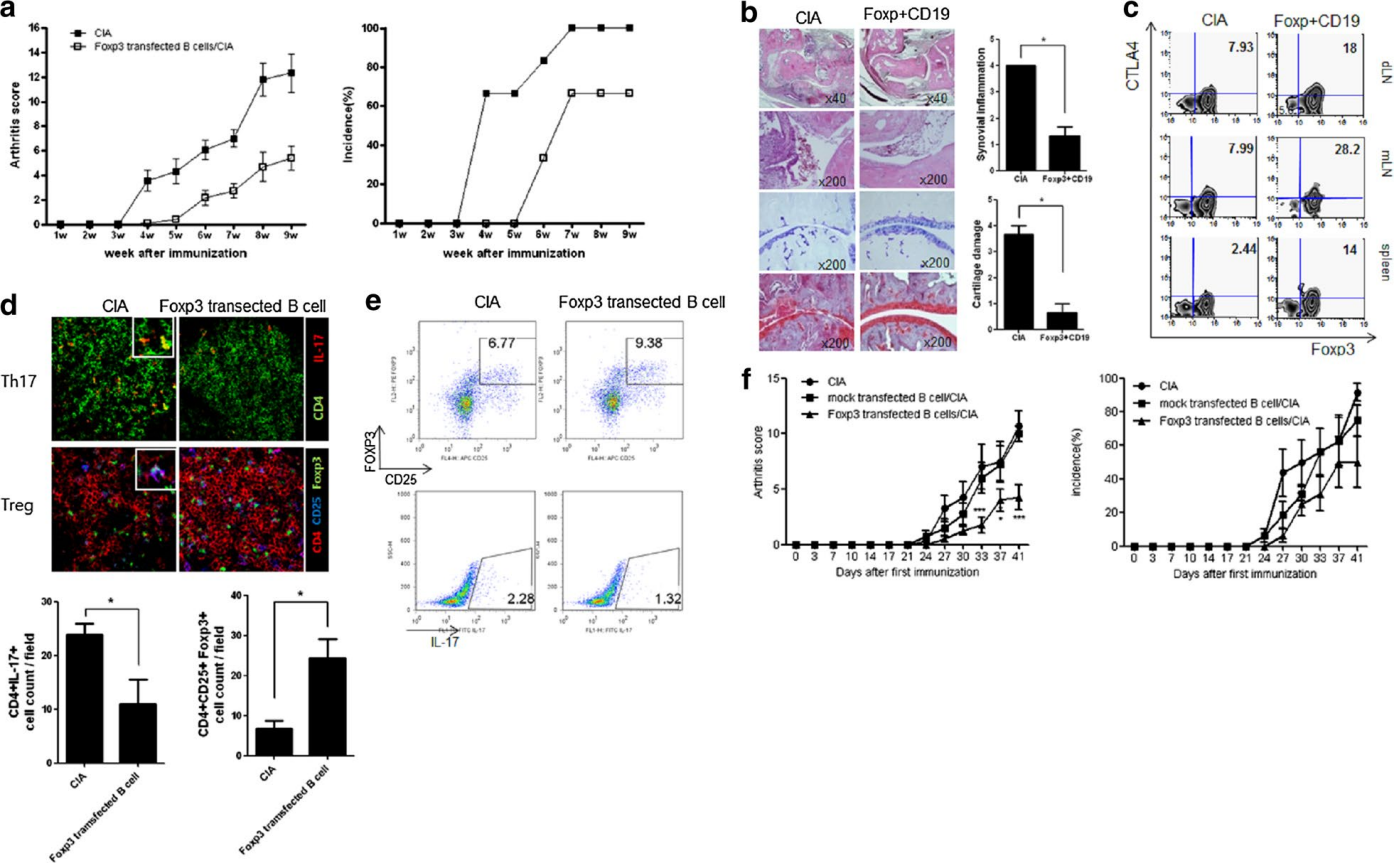
Adoptive transfer of Foxp3-transfected CD19^+^ B cells protects from CIA. Transfected B cells (1 × 10^7^) with Foxp3 vector were injected i.v. into CIA mice (each group n = 10) on days 7 and 28, which had been immunized with CII/CFA. **a**, **b** Evaluation of the severity of arthritis (**a**) by measurement of the increase in paw swelling and histologic examinations (**b**) of the joints of control mice or mice injected with transfected B cells. Representative* photographs* from each group are shown. Original magnifications, ×40, ×200. **c** Percentage of CD4^+^Foxp3^+^CTLA4^+^ Treg cells in the spleens, mesenteric lymph nodes (mLN) and draining lymph nodes (dLN) from control CIA or mice injected Foxp3-transfected CD19^+^ B cells. Cells were stained with CD4-PerCP cy5.5 mAb before permeabilization and stained using Foxp3-FITC and CTLA4-PE mAb. FACS analyses showed that Foxp3^+^CLTA4^+^ cells gated on CD4^+^ T cells. **d**, **e** Spleens from mice of each group were stained with anti-CD4 (*green*) and anti-IL-17 (*red*) (*upper image*) or anti-CD4 (*red*), anti-CD25 (*blue*), and anti-Foxp3 (*green*) (*lower image*) antibodies. Populations of CD4^+^CD25^+^Foxp3^+^ and CD4^+^IL-17^+^ T cells were analyzed using confocal laser microscopy and Flow cytometry. **f** Mock and Foxp3 transfected B cells (1 × 10^7^) were injected i.v. into CIA mice (each group n = 10) on days 7 and 28, which had been immunized with CII/CFA. Original magnification, ×400. Data are expressed as the means ± SD. *p < 0.05 compared with control CIA mice

RA is an antigen-specific T cell-mediated autoimmune disease. Therefore, we tested whether Foxp3-transfected CD19^+^ B cells could ameliorate the development of arthritis in a CIA model. Splenic CD19^+^ B cells transfected with Foxp3 were transferred to DBA/1J mice on days 7 and 28 after the first immunization. Disease progression was monitored. In control mice, clinical disease was apparent on day 21 after the first immunization and became steadily worse until the end of the experiment (Fig. 5a). Mice receiving Foxp3-tranfected B cells had a lower clinical score of the affected arthritic joints and a delay in the development of arthritis compared to the CIA mice (Fig. 5a, f). Consistent with clinical observations, both the cellular infiltrate within the synovium and the level of cartilage degeneration were substantially lower in the group transfected with Foxp3. CIA mice showed a marked synovial thickening, pannus formation, and cartilage and bone destruction (Fig. 5b). Interestingly, numbers of CD4^+^Foxp3^+^CTLA4^+^ Treg cells were increased in the lymph nodes and the spleens of Foxp3-transfected mice compared to CIA mice (Fig. 5c). We also observed that Th17 cell numbers increased in the spleen of CIA mice. In contrast, a larger population of Foxp3^+^ Treg cells and a smaller population of Th17 cells were observed in mice receiving Foxp3-tranfected B cells (Fig. 5d, e). Moreover, Foxp3 transfected B cells reduced significantly CIA development compared to mock transfected B cells. These data suggest that Foxp3-expressing CD19^+^ B cells are protective against arthritis development.

## Discussion

Foxp3 is the most specific marker of regulatory T cells [31, 37]. Up to now, Foxp3 expression has been found only in CD4^+^ T cells and in some tumor cell lines [38]. Transformation of B cells with EBV was reported to express Foxp3, although normal B cells do not express Foxp3 [38]. In this study, we showed that Foxp3-expressing CD19^+^ B cells exist in both normal and autoimmune arthritis mice. The origins of these B cells and how they develop remain unclear. Given the fact that Foxp3^+^CD19^+^ B cells constitute only a small fraction of B cells, transfection of Foxp3 into B cells provides a useful method to generate regulatory B cells in vitro. *In vitro*-generated regulatory B cells can be utilized to inhibit the progression of ongoing autoimmune processes. Our data suggest that transfection of Foxp3 into CD19^+^ B cells induced functional regulatory T cells and suppressed effector T cell proliferation. As a result, Foxp3-infected B cells delayed the onset of arthritis and suppressed its severity in CIA mice.

Regulatory B cell subsets are recognized as an important component of the immune system. Several reports have shown that regulatory B cells influence T cell activation and inflammatory responses through the secretion of IL-10 [10]. Several phenotypes of regulatory B cells have been described. Peritoneal CD5^+^ B-1a cells are known to produce IL-10 [4, 17]. CD5^+^ B cells also produce IL-10 upon IL-12 stimulation [39]. Splenic B cells with a CD21^+^ CD23^−^ MZ phenotype from lupus mice produce IL-10 in response to CpG stimulation [40]. Splenic CD1d^hi^CD21^+^ CD23^+^IgM^+^ B cells with a T2-MZP phenotype also produced IL-10 and inhibited the development of CIA [18]. IL-10-producing CD1d^hi^CD5^+^ regulatory B cell subset showed a suppressive effect against autoimmune encephalitis [20]. Recently, a novel subset of IL-10-producing regulatory B cells, distinct from MZ or B-1a cells, was discovered in the intestine and identified as CD5^−^ CD11b^−^ CD21^+^B cells [41]. Regulatory B cells have been demonstrated to exert immunosuppressive functions by inducing Tregs or skewing the cytokine profile of effector T cells toward an immunosuppressive phenotype [42, 43].

Transcription factors play important roles in the development and lineage commitment of lymphocytes. Little is known about the transcriptional factors that regulate the generation of regulatory B cells. Foxp3 is necessary and sufficient for Treg generation and function [44]. Also, Foxp3 expression in T cells is known to be restricted. In our study, the existence of Foxp3^+^ Bregs was demonstrated in mice arthritis model. Foxp3^+^ may play a role in the generation of Bregs, and the over-expression of Foxp3 in B cells induced regulatory effects.

The BCR plays an important role in the development and proliferation of pre-B and B cells [45, 46]. Similarly, Foxp3^+^ regulatory T cell differentiation and function in the periphery is also dependent on suboptimal TCR stimulation [47]. Our results revealed that expression of Foxp3 is induced after BCR stimulation by anti-IgM or LPS. The frequency of splenic Foxp3^+^CD19^+^ B cells was significantly lower in WT than CIA mice. Up-regulation of Foxp3 in B cells of CIA mice may be a consequence of normal B cell activation under the influence of inflammatory conditions. Also, stimulation of Foxp3^+^CD19^+^ B cells with either LPS or anti-IgM, increased their suppressor activity. Although BCR stimulation induced Foxp3, maximal and sustained Foxp3 expression may require additional stimulation with ligands such as CD40 and LPS. Increased Breg cells in CIA animal may rouse the question why these Breg cells cannot protect host from arthritis. We speculate that increased Breg cells are not sufficient in number or function to suppress over-activated effector T cells in arthritis animal model in vivo. There are also other possibilities like increased apoptosis of Breg cells in inflammatory conditions. Further studies may be needed to prove this hypothesis.

Interestingly, our data showed that adoptive transfer of Foxp3^+^CD19^+^ B cells-increased the number of Foxp3^+^ Tregs in vivo. Vallerskog et al. [48] reported that Foxp3^+^ T cell numbers were significantly increased in peripheral blood of rituximab-infused SLE patients. Alteration of T cell populations may be important in the B cell depletion therapy used for autoimmune diseases. Our results showed that Foxp3^+^ Bregs modulated a T cell population. The interaction between B and T cells will likely be important in the treatment of arthritis and other autoimmune diseases.

The mechanism how FoxP3 B cells are working in vivo needs be elucidated. We observed that Foxp3 B cells secreted large amount of IL-10 and TGF-β (data is not shown). IL-10 or TGF-β producing B cells have been reported to play important role in regulatory function [49–51]. IL-10 inhibits pro-inflammatory cytokine and supports regulatory T cell differentiation so play pivotal role of immune tolerance.

## Conclusion

In summary, we report a significant function of regulatory B cells expressing Foxp3 in CIA. The regulatory effect of Foxp3^+^ B cell showed contact-dependent. Foxp3^+^ B cells successfully suppress arthritis and induced the Treg cell population. Identification of mechanism related to the induction of Treg cells remains an important area for future study. Therapy using Foxp3^+^ B cells is considered as an intriguing new intervention to approach various autoimmune/inflammatory diseases.

## Abbreviations

Treg: regulatory T cells
CIA: collagen-induced arthritis
RA: rheumatoid arthritis
RF: rheumatoid factor
IPEX: immunodysregulation, polyendocrinopathy, enteropathy, and X-linked
CTLA4: cytotoxic T lymphocyte antigen 4
GITR: glucocorticoid-induced TNF receptor-related protein
ORF: open reading frame
