# Erratum to: Amelioration of autoimmune arthritis by adoptive transfer of Foxp3-expressing regulatory B cells is associated with the Treg/Th17 cell balance

**DOI:** 10.1186/s12967-016-0980-z

**Published:** 2016-07-26

**Authors:** Mi-Kyung Park, Young Ok Jung, Seon-Yeong Lee, Seung Hoon Lee, Yu-Jung Heo, Eun-Kyung Kim, Hye-Jwa Oh, Young-Mee Moon, Hye-Jin Son, Min-Jung Park, Sung-Hwan Park, Ho-Youn Kim, Mi-La Cho, Jun-Ki Min

**Affiliations:** 1 The Rheumatism Research Center, Catholic Research Institute of Medical Science, The Catholic University of Korea, 505 Banpo-dong, Seocho-gu, Seoul, 137-040 South Korea; 2 Division of Rheumatology, Department of Internal Medicine, Hallym University Kang-Nam Sacred Heart Hospital, Seoul, South Korea; 3 Division of Rheumatology, Department of Internal Medicine, College of Medicine, The Catholic University of Korea, Seoul, South Korea; 4 Bucheon St. Mary’s Hospital, Division of Rheumatology, Department of Internal Medicine, College of Medicine, The Catholic University of Korea, 327 Sosa-ro, Wonmi-gu, Bucheon, Gyeonggi-do 420-717 South Korea; 5 Division of Rheumatology, Department of Internal Medicine, College of Medicine, Holy Family Hospital, Rheumatism Research Center (RhRC), Catholic Research Institute of Medical Science, The Catholic University of Korea, Seoul, South Korea

## Erratum to: J Transl Med (2016) 14:191 DOI 10.1186/s12967-016-0940-7

Unfortunately, the original version of this article [1] contained errors. The author’s names were incorrectly marked up and display incorrectly on PubMed. Additionally, the funding information was incorrect. The correct author list and funding section can be found below. The author names and funding section have been corrected in the original article [1] and is also included correctly in this erratum.

## References

[1] Park M-K, Jung YO, Lee S-Y, Lee SH, Heo Y-J, Kim E-K, Oh H-J, Moon Y-M, Son H-J, Park M-J, Park S-H, Kim H-Y, Cho M-L, Min J-K (2016). Amelioration of autoimmune arthritis by adoptive transfer of Foxp3-expressing regulatory B cells is associated with the Treg/Th17 cell balance. J Transl Med.

